# Magnetic resonance Adenosine perfusion imaging as Gatekeeper of invasive coronary intervention (MAGnet): study protocol for a randomized controlled trial

**DOI:** 10.1186/s13063-017-2101-6

**Published:** 2017-07-28

**Authors:** Dominik Buckert, Simon Witzel, Maciej Cieslik, Raid Tibi, Wolfgang Rottbauer, Peter Bernhardt

**Affiliations:** 1 Department of Internal Medicine II, University of Ulm, Albert-Einstein-Allee 23, 89081 Ulm, Germany; 2 Heart Clinic Ulm, Ulm, Germany

**Keywords:** Stable coronary artery disease, Cardiac magnetic resonance imaging, Coronary angiography, Myocardial revascularization, Outcomes and prognosis, Quality of life

## Abstract

**Background:**

Current guidelines for the diagnosis and management of patients with stable coronary artery disease (CAD) recommend functional stress testing for risk stratification prior to revascularization procedures. Cardiac magnetic resonance imaging (CMR) is a modality of choice for stress testing because of its capability to detect myocardial ischemia sensitively and specifically. Nevertheless, evidence from randomized trials evaluating a CMR-based management of stable CAD patients in comparison to a more common angiography-based approach still is limited.

**Methods/design:**

Patients presenting themselves with symptoms indicating a stable CAD and a class I or IIa indication for diagnostic coronary angiography are prospectively screened and enrolled in the study. All subjects receive a basic cardiological work-up and guideline-directed medical therapy. A 1:1 randomization in two groups is being performed. Patients in group 1 undergo diagnostic coronary angiography and subsequent revascularization according to current guidelines. Subjects in group 2 undergo adenosine stress CMR and in case of myocardial ischemia are sent to coronary angiography. Follow-up is planned for 3 years. During this time, the number of primary endpoints (defined as cardiac death and non-fatal myocardial infarction) and unplanned invasive procedures will be documented. Furthermore, symptom burden and quality of life will be assessed by use of the Seattle Angina Questionnaire. Sample size is calculated to prove non-inferiority of the CMR-based approach.

**Discussion:**

In case this study is able to accomplish its aim to prove non-inferiority of the CMR-based management in patients with stable CAD; the importance of this emerging modality may further increase.

**Trial registration:**

ClinicalTrials.gov, identifier: NCT02580851. Registered on 14 October 2015. Unique Protocol ID: 237/11

**Electronic supplementary material:**

The online version of this article (doi:10.1186/s13063-017-2101-6) contains supplementary material, which is available to authorized users.

## Background

Current guidelines for the diagnosis and management of patients with stable coronary artery disease (CAD) recommend – besides thorough history and physical examination – proper risk stratification and non-invasive detection of myocardial ischemia prior to invasive therapy [1, 2]. The detection or exclusion of moderate to severe reversible myocardial ischemia is a crucial part of the work-up process. When ischemia is present, patients are designated to a high-risk group with a high probability of having a prognostic benefit from revascularization procedures [3–5]. Patients without ischemia do not seem to benefit from revascularization over optimal medical therapy, at least with regard to prognosis [6]. This emphasizes the need for functional testing prior to therapeutic decisions.

Adenosine perfusion cardiac magnetic resonance imaging (CMR) is an imaging modality which is able to reliably detect reversible myocardial ischemia and thus plays an increasing role in the diagnosis and risk stratification of patients with suspected or known CAD [7–11]. Though CMR, therefore, is highly recommended in the diagnostic work-up of stable CAD patients, there is only one study evaluating a CMR-driven approach in patient management with regard to the occurrence of major cardiac events [12].

Invasive coronary angiography still is considered to be the “gold-standard” for the diagnosis of CAD, though it exhibits several limitations and shortcomings. Multiple studies have documented the significant interobserver variability in the grading of coronary artery stenosis, as well as the frequent occurrence of under- and overestimation of hemodynamic relevance [13, 14]. One has to conclude that coronary angiography may provide anatomical information but is not the modality of choice concerning the detection of myocardial ischemia. This is of special interest, as there is a reported frequency of complications due to diagnostic coronary angiographies of about 1.5% [15]. Nevertheless, coronary angiography remains the most often performed diagnostic test in the setting of stable CAD, with more than one half of elective percutaneous coronary interventions (PCI) done without previous stress testing [16].

The objective of our study is to show that a CMR-based conservative or invasive management of patients with suspected or known CAD is not inferior with regard to major cardiac endpoints and quality of life in comparison to a – more conventional – coronary angiography-based approach. We assume that a significant number of diagnostic coronary angiographies and PCI could be spared without decrease in patient safety and comfort. Therefore, we designed this single-center, open-label, randomized controlled trial to test for non-inferiority of the two diagnostic groups.

## Methods/design

### General information

This study has been approved by the local Ethics Committee of our university (Ethikkommission der Universität Ulm, Chairman Prof. Dr. Oliver Zolk, reference number 237/11, Additional file 1). It has been partially funded by Guerbet Germany, Sulzbach, Germany (see Additional file 2 for funding contract). The funder does not have any influence concerning study design, data collection, analysis and the decision to submit the report. Other financial expenses and the involved personal are provided by our own institution.

As our study is an investigator-initiated, single-center trial (academic hospital, Germany) there is no coordinating center or steering committee. Patient enrollment, endpoint adjudication and data management will be carried out by the authors of this protocol independently from the sponsor. To monitor the conduction of the trial, the members of the Investigator Committee meet on a regular basis and exceptionally when concerns arise.

Participants are covered by the academic hospital in case harm should occur in the context of this trial. Provisions are not intended.

The study is registered at ClinicalTrials.gov (identifier: NCT02580851). Protocol modification will be communicated to the ethics committee and will be documented at ClinicalTrials.gov. The adapted Standard Protocol Items: Recommendations for Interventional Trials (SPIRIT) Checklist is attached as Additional file 3.

After completion of the trial, publication of the results is intended in a peer-reviewed scientific journal. Publication will only be carried by the authors of this manuscript without the help of professional writers. Granting full access to the protocol or participant-level dataset is not intended.

### Patient recruitment

Generally, informed consent will be obtained by the investigators after personal elucidation from all participants prior to enrollment in the study. All patients presenting themselves in the outpatient clinic of our university hospital for the evaluation of symptoms indicating a stable CAD (e.g., exercise-related angina pectoris or dyspnea) will be screened prospectively. Patients at intermediate to high risk (according to recommended risk scores) and a class I or IIa indication for diagnostic coronary angiography (Additional file 4: Table S1) will be considered eligible unless they exhibit the following exclusion criteria: unstable angina pectoris, cardiac or respiratory instability, contraindication for CMR examination [17], known allergy to gadolinium-based contrast agents, impaired renal function (glomerular filtration rate <30 ml/min), contraindication or allergy to adenosine, pregnancy, age below18 years and inability to give written informed consent. It is irrelevant whether a CAD has already been diagnosed at the time of first presentation.

### Protocol

All patients receive thorough history-taking, physical examination and a basic cardiological work-up including rest electrocardiogram, treadmill testing and echocardiography. Further prediagnostics (e.g., stress-echocardiography) is allowed and the respective results will be documented. According to general recommendations, risk stratification scores for each individual are calculated [18, 19]. In order to assess symptom burden and quality of life, the standardized Seattle Angina Questionnaire (SAQ) is carried out in each subject [20, 21]. All patients receive guideline-directed medical therapy (GDMT) [1].

Patients are randomized in two groups in a 1:1 fashion (blocked computer-generated random numbers). The allocation sequence is only available to a designated study nurse who will be phoned in case of an allocation. The trial is designed in an open-label fashion, i.e., there is no blinding after the initial allocation.

In group 1, patients directly undergo diagnostic coronary angiography. A PCI is performed according to current guidelines in case of ≥70% stenosis in a coronary vessel with ≥2-mm diameter [1, 2] or hemodynamically relevant stenoses in fractional flow reserve (FFR) testing.

Subjects in group 2 initially receive adenosine perfusion CMR for functional testing. The examination is conducted on a 3.0-Tesla whole-body scanner with a 32-channel phased-array cardiac receiver coil according to a well-established standard protocol [22–24]. In case reversible ischemia can be detected, subjects are sent to coronary angiography and PCI afterwards. Additional file 5: Figure S1 provides the study flowchart.

In case of progressive or insufficient symptom control, patients of group 2 (CMR group) are also evaluated for an invasive therapeutic approach. The following procedures will be documented.

There are no other diagnostic or therapeutic interventions planned or prohibited in the setting of this trial.

### Follow-up

Adherence will be achieved by regular contact between the study personal and the participants. Follow-up information is gathered on a yearly basis after enrollment by outpatient clinic visits and by telephone interviews of patients and their general practitioners. Any reported adverse event will be reported and its significance evaluated by the investigators. At this time point, interim analyses will be performed by the investigators. The study will be stopped by the investigators if a safety concern arises that prohibits the continuation of the trial.

The primary endpoint is defined as cardiac death (defined as in [25]) and non-fatal myocardial infarction according to the current universal definition [26]. Any diagnostic or interventional coronary procedure that has not been scheduled at the time of the initial diagnostic work-up will be recorded as an unplanned procedure. Symptom burden and quality of life are assessed by SAQ each year. A follow-up period of 3 years is planned for each patient. Standardized follow-up forms have been established and are available at our institution. If consent is withdrawn by a participant, the data that has already been collected will be removed from further analysis.

**Fig. 1:**
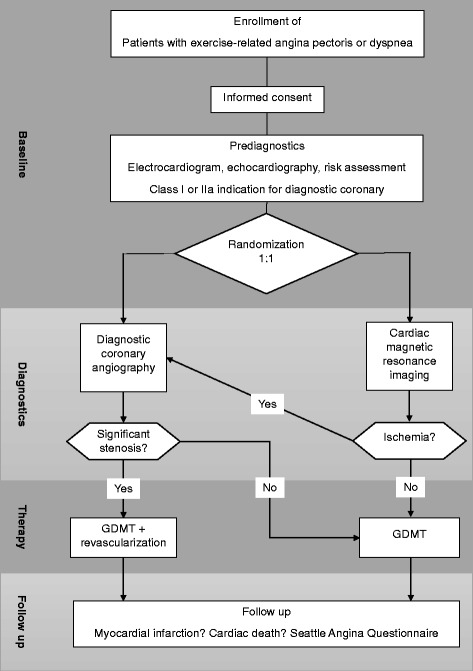
SPIRIT figure illustrating the study schedule

Figure 1 shows the study schedule according to the populated SPIRIT figure.

### Sample size prediction/statistics

It has been shown that the annual event rate for the defined primary endpoint is 6.3% in case of a pathological and 1.0% in case of a normal CMR examination [27]. Retrospective analysis of our data revealed a 30% rate of pathological CMR examinations during the last years, thus the expected annual event rate would be 2.59% for the whole primary CMR group. In patients primarily undergoing coronary angiography, pooled annual event rates have been shown to be about 6% [6, 28]. For the sample size prediction, a Fisher’s exact test with a power of 80% and a *p* value indicating significance of < .05 was used. The study was designed to prove non-inferiority with a defined non-inferiority margin of 1% within a 3-year follow-up period. This lead to a predicted sample size of 90 participants per group. Taking dropout rate and safety margin into account, we plan to enroll 100 patients into each group. Annual interim analyses will be performed to evaluate the need for further enrollment in case of insufficient event rates for an appropriate statistical analysis.

The following statistical tests will be used after completion of follow-up: to test the relationship between categorical classification factors, the chi-squared test will be applied. Continuous variables will be tested for normal distribution by the D’Agostino-Pearson test. In case of a normal distribution, variables will be reported as mean ± standard deviation and a two-tailed *t* test will be applied for comparison. Variables without normal distribution will be reported as median with percentiles and the Mann-Whitney rank-sum test will be used for comparison. As mentioned above, Fisher’s exact test will be applied to test for non-inferiority of the event rates of both groups. Overall, a *p* value ≤ .05 will be judged significant.

Data management will only be carried out by trained personal. The database is stored on protected servers of the academic hospital.

## Discussion

In contrast to the recommendations given in current guidelines, diagnostic coronary angiography remains the most often applied modality in the evaluation of patients with stable CAD [1, 2]. In consequence, a high rate of angiographies without detection of obstructive CAD results [13, 15]. This is of special interest, as there is a remarkable frequency of complications associated with this procedure [15]. On the other hand, many diagnostic angiographies are followed by interventional revascularization procedures without prior functional assessment of visually detected coronary stenosis [16]. Taken together, expansion of invasive diagnostic and therapeutic procedures lead to a growing economic burden stressing health care systems without evidence for ameliorated patient care [6].

CMR is an important diagnostic modality in the growing arsenal of cardiology. Its ability to reliably and simultaneously evaluate global cardiac function and myocardial ischemia has been proven previously [11]. In particular, the high negative predictive value of a normal CMR examination with regard to the occurrence of major cardiac endpoints predisposes this modality to act as gatekeeper prior to revascularization strategies [29].

To the best of our knowledge, there is only one study prospectively randomizing patients to a CMR-driven versus an angiography-driven management strategy [12]: the MR-INFORM trial was a randomized multicenter study comparing a FFR- versus a CMR-based management approach of stable CAD patients. Non-inferiority of the CMR-based strategy could be demonstrated within a 12-month follow-up. In the present trial, a follow-up for up to 3 years is planned for every patient, thus providing information on long-term outcomes for each management strategy. Moreover, besides major clinical endpoints, symptom burden and quality of life will also be assessed. This is of special interest since clinical decision-making always should be based on patient comfort and satisfaction.

If the present study can accomplish its aim to prove non-inferiority of the CMR-based approach in the management of patients with stable CAD, its importance may further increase. This might result in a reduction of adverse events associated with coronary angiographies and revascularization procedures. This trial, therefore, aims to contribute important evidence with regard to the management of stable CAD patients.

## Trial status

At the time of submission, patient recruitment is ongoing.

## Additional files

Additional file 1: Ethics approval document. (JPG 766 kb)

Additional file 2: Funding contract. (PDF 1 mb)

Additional file 3: SPIRIT Checklist. (DOC 118 kb)

Additional file 4: **Table S1**. Indications for coronary angiography. (DOCX 11 kb)

Additional file 5: **Figure S1**. Flowchart depicting study protocol. GDMT – guideline-directed medical therapy. (DOCX 31 kb)

## Abbreviations

CAD: Coronary artery disease
CMR: Cardiac magnetic resonance imaging
FFR: Fractional flow reserve
GDMT: Guideline-directed medical therapy
PCI: Percutaneous coronary intervention
