# Supplementary figures and images for: Magnetic resonance Adenosine perfusion imaging as Gatekeeper of invasive coronary intervention (MAGnet): study protocol for a randomized controlled trial
Source: Trials. 2017 Jul 28;18:358. doi: 10.1186/s13063-017-2101-6 (PMC5534045; doi:10.1186/s13063-017-2101-6)

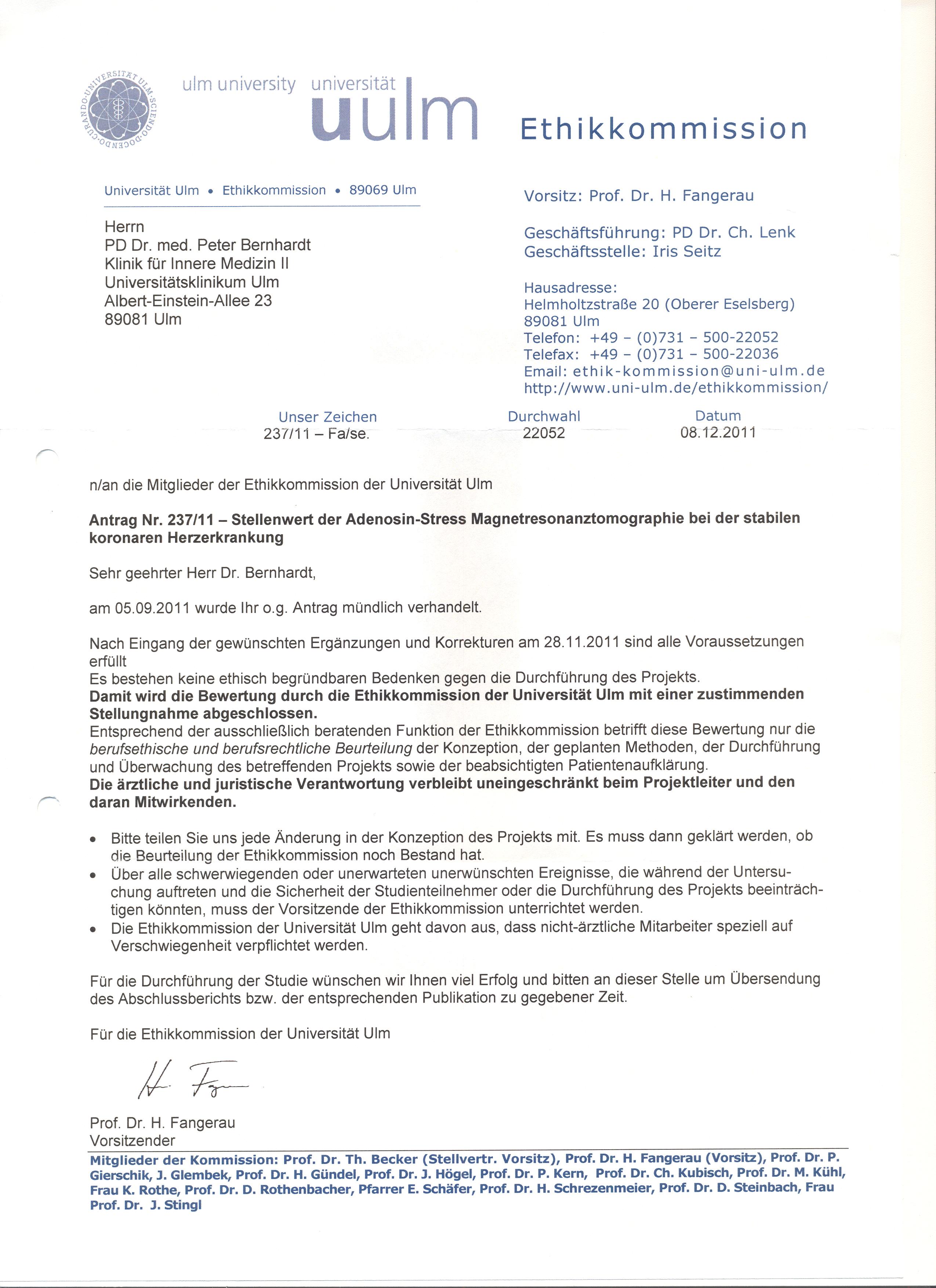

Supplement: Supplementary file 1 — Ethics approval document. (JPG 766 kb) [file 13063_2017_2101_MOESM1_ESM.jpg]
