# Supplementary material for: Magnetic resonance Adenosine perfusion imaging as Gatekeeper of invasive coronary intervention (MAGnet): study protocol for a randomized controlled trial
Source: Trials. 2017 Jul 28;18:358. doi: 10.1186/s13063-017-2101-6 (PMC5534045; doi:10.1186/s13063-017-2101-6)
Supplement: Supplementary file 2 — Funding contract. (PDF 1 mb) [file 13063_2017_2101_MOESM2_ESM.pdf]

Contract for the ***financial support of a clinical trial (investigator-initiated study)*** *with the title:*

MAGnet trial - Magnetic Resonance Adenosine Perfusion Imaging as Gatekeeper of invasive coronary intervention

Between

GUERBET, BP 57400, 95943 Roissy CdG Cedex, France

- referred to below as 'Company' -

and

University of Ulm represented by the authorized person, Prof. Dr. Thomas Seufferlein, Acting Chief medical Director
Executing Department: Department of Internal Medicine II
Medical Director: Prof. Dr. Wolfgang Rottbauer
Address: Albert-Einstein-Allee 23, 89081 Ulm, Germany

On behalf of the University of Ulm, Faculty of Medicine

- referred to below as the 'Sponsor' -

with **the principal investigator Prof. Dr. P. Bernhardt**

- referred to below as the 'Investigator' -

Preamble
Sponsor and Investigator are planning to perform a clinical trial entitled:

MAGnet trial - Magnetic Resonance Adenosine Perfusion Imaging as Gatekeeper of invasive coronary intervention

For the avoidance of doubt this clinical trial is neither a clinical trial according to the German Drug Act (Arzneimittelgesetz) nor to the German Medical Device Act (Medizinproduktegesetz). The competent Ethics Commission has given his positive judgement.

The Sponsor wishes, in order to perform the Study, to have a financial support
In return of such financial support, Sponsor asked the Company whether it would be interested to have access and use the results of the Study.

Consequently the Parties hereby agree

Article 1. Object:

1.1 Sponsor and Investigator hereby agree to that the Company will have the rights to use and reproduce the non-protectable results related to the Study (hereinafter the Rights). These Rights include without any limitation the right to use them worldwide and by any mean.
This right to use the Rights does not prevent Sponsor and Investigator to make any publication related to the results of the Study.

Company's initials	Investigator's initials	Sponsor's initials

1.2 Company shall grant Sponsor
a subvention amount of **€ 60,000** (sixty thousand euros) (the Subvention).

Article 2. Responsibility:

The Study shall be carried out on the initiative and under the sole and exclusive responsibility of the Sponsor and Investigator. Thus:

- Study shall be conducted according to the requirements of the recommendations of the ICH GCP (Harmonized Tripartite Guideline for Good Clinical Practice) and the current version of the Helsinki Declaration
- The prerequisite for initiating the Study is the approval of the Ethics Committee and Competent Authorities of the Sponsor
- The Sponsor and the Investigator parties shall warrant that they will maintain sufficient fund capability or insurance in types and amounts reasonably adequate to cover any liabilities arising out of their respective obligations hereunder In no event the Company is either the contract-giver or the sponsor of the Study as defined in the ICH-GCP Guidelines. Therefore the Company has no responsibility for the conduct of the Study. Sponsor and Investigator agree to and hereby indemnify and hold Company harmless from and against all third-party claims, damages, losses, costs and expenses, which may incur as the result of the death or injury to persons or damage resulting from the Study not being in compliance with applicable regulations or against any third-party claims, damages, losses, costs and expenses incurred as a result of Sponsor or Investigator liabilities.
- The Investigator will report without delay (i.e. within 24 hours) to the Company (Fax number: + 33145916770 or e-mail: pharmacovigilance.headquarters@guerbet-group.com) all serious adverse reactions occurring in the course of the Study so that the Company can fulfill its obligations regarding the periodical review and evaluation of relevant safety information about its products. The Investigator agrees to provide further information on a specific case to the Company upon request. In parallel, the Company will share with the Investigator any new safety information that may have an impact on the management of the Study and/or on the patients' safety. However, this exchange of safety data between both parties does not preclude the Investigator from his regulatory obligations to report all serious adverse reactions to the relevant competent authority.
- The investigator will report to IRB for all the information related with subject's safety or any influence to conduct the trial if it is found out during monitoring or review the data and result within 2 years after trial termination.

Article 3. Payment of the Subvention:

Company will pay Subvention in third instalments:

The first instalment of **€ 30000** (Thirty thousand euros) is due 30 days after signing the contract.
The second instalment of **€ 15000** (Fifteen thousand euros) is due 30 days after the completion of 100% Case report Forms (CRF) of the planned patient population.
The third instalment of **€ 15000** (Fifteen thousand euros) is due 30 days after the reception of manuscript suitable for publication.

Should the contractual payments to the Sponsor be subject to VAT, the Sponsor is entitled to request the legal VAT, in addition to the payments agreed upon in this contract, if the Company receives an invoice with special mention of the VAT. In this respect the company forgoes the claim for a statute of limitations.

All payments by the Company shall be made to Sponsor account of 'University Hospital of Ulm'

Company's initials	Investigator's initials	Sponsor's initials

Address of Account holder:	Albert-Einstein-Allee 29; 89081 Ulm, Germany
Name of bank:	Sparkasse Ulm
Address of bank:	Neue Straße 66, 89073 Ulm, Germany
Account number:	106 478
Bank account IBAN:	DE 166 305 0000 0000 106 478
SWIFT (BIC):	SOLADES1ULM
University Hospital VAT number:	DE 147 040 060
Project account (sub ledger):	D 4945-503/14

Article 4. Conditions of use of the Rights.

The Sponsor and the Investigator grant to the Company a free access on approved protocol, CRF, ICF and amendment and progress report for recruitment and data collection during the trial conducting period and use the non-protectable results after trial completion.

All results (whether protectable or not) obtained during the Study remain the sole property of the Sponsor but Company will have the right to use the non-protectable results as set forth in Article 1 here above.

The reproduction of trial results will not distorted or misleading in this respect.

The Sponsor represents and guarantees the Company hereby that until their publication, if any, the results of the Study will not be disclosed to any third party, and besides the Sponsor only the Company will have at any time the right to use in part or in whole the non-protectable results.

In the event that inventions arise in connection with the performance of the Study, such inventions shall be due to the Sponsor with due regard to the German Employee's Invention Law (ArbnErfG). If the Sponsor will register and /or maintain an industrial property right relating to this invention, the Parties can agree on rights of use for the Company. Details thereto shall be agreed among the Parties hereto in an additional agreement, specifying reasonable and acceptable market terms.

Use of the name of the Sponsor by the Company, apart from the end-of-study report or the usual mention of authors (e.g. on publicity materials), shall require prior written approval by the Sponsor.
Should the Sponsor plan to publish the results of the Study, he will forward to the Company a draft of the publication at least one (1) month before its submission for publication in order to give the opportunity to the Company to make comments.
Changes requested by the Company shall be taken into account, if this does not affect the scientific character or neutrality of the planned publication.

Article 5. Confidentiality

During the Study period and during a five-year period after the termination of the Study, Parties shall maintain the confidentiality of any information and data provided by the other Party or relating to products and businesses, including but not limited to financial and other business information, product samples, formulas, certificates of analysis, manufacturing processes, standard operating procedures, pricing information, personnel information, specifications, drawings, schematics and other technical, customer and product development plans, forecasts, related to the Study, hereafter the "Confidential Information". "Confidential Information" shall also include, whether in oral, visual, written, graphic or electronic form: (i) all information, work papers, analyses, compilations, projections, studies, memoranda, notes, reports, summaries, documents, correspondence, facts or other materials (including copies thereof) derived, learned or prepared by any party in connection with

the Purpose and marked as confidential; (ii) any information the nature of which is such that a reasonable person would consider the information to be confidential or proprietary.

The obligation of confidentiality does not apply to information that was demonstrably known by the receiving partner before communication, or was independently obtained or legally acquired, or if this information is inherent in the current state of the art.
The obligation of confidentiality shall not apply to Confidential Information that Sponsor and Investigator have been expressly authorized to disclose by the Company.

Any authorized disclosure of the Results or the Study shall mention the Company as the financial support of the Study.

Article 6. Principle of separation.

The Parties agree that the contract shall have no effect on the trading transactions of the Sponsor, particularly purchasing transactions / pricing, and no expectations shall arise in this respect. The Sponsor, the Investigator and employees of the Sponsor shall receive no unrelated benefits from carrying out the Study apart from the financial support mentioned here above.

Article 7. Relations between the industry and the scientific health worlds

According to the national regulations of Germany, the Investigator will have to disclose his/ her relationship with Guerbet to his/her Health Authorities, whenever he/she writes or speaks in public on the subject matter to which the Contract refers. The same obligation also applies in the event that Guerbet employs physicians on a part-time basis who are also practicing medical professionals.

Article 8. Miscellaneous

Changes or additions to this contract must be in writing. Collateral agreements, verbal or otherwise, have not been made. This is also valid for this regulation.

Should individual provisions of this contract prove to be ineffective, the validity of the other provisions shall not be affected. In place of the ineffective provision, the next best arrangement shall be valid, as wished by the contracting partners or as they would have wished if they had been aware of the inefficacy of the provision.

Governing Law / Jurisdictional Venue

This Contract shall be subject to the laws of Germany. Any conflict of laws provisions shall not apply. This Contract shall be construed according to German Law. In the event that there is any conflict between the English legal meaning and the German legal meaning of this Contract or any part hereof, the German legal meaning shall prevail.

Jurisdictional venue shall be in the court of Ulm, Germany.

Company's initials	Investigator's initials	Sponsor's initials

For the Company: **Guerbet Headquarters, Paris in France**

Signature ______________________ Date 23 April 2015

Pierre Desche, MD

Head of Development, Medical & Regulatory Affairs

Guerbet Ltd.

Management/authorised representative of the Company

For University Hospital of Ulm in Germany

University Hospital of Ulm
[illegible]
Legal Department
89070 Ulm

Signature i.V. ______________________ Date 26.03.2015

Prof. Dr. Thomas Seufferlein
Acting Chief Medical Director

Read and acknowledged:

Signature ______________________ Date 18 MARCH 2015
Prof. Dr. Wolfgang Rottbauer
Medical Director

Signature ______________________ Date 18-MAR-2015
Prof. Dr. Peter Bernhardt
Principal Investigator

Company's initials	Investigator's initials	Sponsor's initials
