# Supplementary material for: Magnetic resonance Adenosine perfusion imaging as Gatekeeper of invasive coronary intervention (MAGnet): study protocol for a randomized controlled trial
Source: Trials. 2017 Jul 28;18:358. doi: 10.1186/s13063-017-2101-6 (PMC5534045; doi:10.1186/s13063-017-2101-6)
Supplement: Supplementary file 4 — Table S1. Indications for coronary angiography. (DOCX 11 kb) [file 13063_2017_2101_MOESM4_ESM.docx]

Additional file 4: Table S1

Indications for diagnostic coronary angiography

Class I

(1) Patients determined to be at high risk for adverse outcome on the basis of non-invasive testing even if they present with mild or moderate symptoms of

angina (level of evidence B)

(2) Severe stable angina (Class 3 of Canadian Cardiovascular Society Classification (CCS), particularly if the symptoms are inadequately responding to

medical treatment (level of evidence B)

(3) Stable angina in patients who are being considered for major non-cardiac surgery, especially vascular surgery (repair of aortic aneurysm, femoral bypass, carotid endarterectomy) with intermediate or high risk features

on non-invasive testing (level of evidence B)

Class IIa

(1) Patients with an inconclusive diagnosis on non-invasive testing, or conflicting results from different noninvasive modalities (level of evidence C)

(2) Patients with a high risk of restenosis after PCI if PCI has been performed in a prognostically important site (level of evidence C)
