# Supplementary material for: Magnetic resonance Adenosine perfusion imaging as Gatekeeper of invasive coronary intervention (MAGnet): study protocol for a randomized controlled trial
Source: Trials. 2017 Jul 28;18:358. doi: 10.1186/s13063-017-2101-6 (PMC5534045; doi:10.1186/s13063-017-2101-6)
Supplement: Supplementary file 5 — Figure S1. Flowchart depicting study protocol. GDMT – guideline-directed medical therapy. (DOCX 31 kb) [file 13063_2017_2101_MOESM5_ESM.docx]

Additional file 5: Figure S1

**Baseline**

Enrollment of

Patients with exercise-related angina pectoris or dyspnea

Informed consent

Prediagnostics

Electrocardiogram, echocardiography, risk assessment

Class I or IIa indication for diagnostic coronary angiography

Randomization 1:1

Diagnostic coronary angiography

Cardiac magnetic resonance imaging

Significant stenosis?

Ischemia?

Yes

GDMT + revascularization

GDMT

Yes

No

No

Follow up

Myocardial infarction? Cardiac death? Seattle Angina Questionnaire

**Diagnostics**

**Therapy**

**Follow up**
